# Prevalence of breakfast skipping among children and adolescents: a cross-sectional population level study

**DOI:** 10.1186/s12887-022-03284-4

**Published:** 2022-04-23

**Authors:** Alanna Sincovich, Hero Moller, Lisa Smithers, Mary Brushe, Zohra S. Lassi, Sally A. Brinkman, Tess Gregory

**Affiliations:** 1 Telethon Kids Institute, the University of Western Australia, Ground Floor, 108 North Terrace, Adelaide, South Australia 5000 Australia; 2 School of Public Health, the University of Adelaide, Level 5, Rundle Mall Plaza, Adelaide, South Australia 5000 Australia; 3 School of Health and Society, University of Wollongong, Northfields Avenue, Wollongong, New South Wales 2522 Australia; 4 Robinson Research Institute, the University of Adelaide, Level 1, Helen Mayo North, 60 Frome Road, Adelaide, South Australia 5005 Australia

**Keywords:** Breakfast skipping, Breakfast consumption, Children and adolescents, School breakfast program, Wellbeing and engagement collection

## Abstract

**Background:**

Interventions to promote breakfast consumption are a popular strategy to address early life inequalities. It is important to understand the epidemiology of children and adolescents who skip breakfast so that interventions and policy can be appropriately considered. This study investigated the prevalence of breakfast skipping among a contemporary, population-wide sample of children and adolescents in Australia.

**Methods:**

Participants were grade 4–12 students (*n* = 71,390, 8–18 years) in South Australian government (public) schools who took part in the 2019 Wellbeing and Engagement Collection. The prevalence of breakfast skipping (never, sometimes, often, or always) was calculated for the overall sample and stratified by gender, school grade, socioeconomic status and geographical remoteness. Multinomial logistic regression analyses were conducted to determine the relative risk ratio of sometimes, often, and always skippers compared with never skippers, according to demographic characteristics.

**Results:**

Overall, 55.0% of students reported never skipping breakfast, 17.4% reported sometimes skipping, 18.0% reported often skipping, and 9.5% reported always skipping breakfast. Skipping breakfast was more prevalent among females, students in senior grades, and those living in socioeconomically disadvantaged and regional and remote areas. Analyses disaggregated by gender revealed that grade level gradients in breakfast skipping were more marked among females compared to males.

**Conclusions:**

Breakfast skipping among children and adolescents appears considerably more prevalent than previous research suggests. Drivers of breakfast skipping across population sub-groups need to be explored to better inform strategies to promote breakfast consumption.

**Supplementary Information:**

The online version contains supplementary material available at 10.1186/s12887-022-03284-4.

## Background

Breakfast consumption is an important aspect of a healthy lifestyle, improving nutrient intake and providing energy for physical and cognitive function [1, 2]. Breakfast skipping among children and adolescents is associated with poorer school attendance and academic performance, reduced wellbeing, and unhealthy dietary and physical activity behaviours [2–4], each of which continue to have adverse effects throughout the life course [5, 6].

Evidence regarding the prevalence of breakfast skipping varies depending on how breakfast consumption is measured and how breakfast skipping is defined. A recent systematic review reporting on the prevalence of breakfast skipping among children and adolescents from 33 countries (*n* = 285,626, aged 2–18 years) concluded that most studies reported between 10–30% of young people skipped breakfast [7]. Commonly reported reasons for breakfast skipping among children and adolescents include those related to a lack of time, enjoyment of breakfast, or feelings of hunger in the morning, and weight control [8]. Evidence suggests that breakfast skipping is most prevalent among females, older children, and adolescents [2, 7, 9, 10]. Further, as with many health-related behaviours, breakfast skipping is socioeconomically patterned and tends to cluster with other unhealthy behaviours such as poor diet, exercise, and sleep habits [2, 9–11]. The extent to which eating breakfast contributes to health, education, and wellbeing disparities between breakfast consumers and breakfast skippers, however, remains unclear.

Research focused on the prevalence of breakfast skipping among children and adolescents in Australia is limited. The 2007 Australian National Children’s Nutrition and Physical Activity Survey was used to explore breakfast skipping among 4487 children aged 2–16 years [12]. Breakfast consumption was measured using two 24-h recalls (caregiver or child/adolescent reported, depending on participant age), with 3.4% of males and 5.4% of females identified as breakfast skippers, having skipped breakfast on both recall days. Research using data from the Longitudinal Study of Australian Children collected in 2008 reported on breakfast skipping among 2280 children aged 8–9 years [13]. Caregiver-reported breakfast consumption was recorded on three occasions, with 10.5% of males and 10.8% of females having skipped breakfast at least once. Most recently, data from the 2011–12 National Nutrition and Physical Activity Survey were used to explore breakfast skipping among 1592 2–17 year olds [14]. Breakfast consumption was measured using two 24-h recalls (caregiver or participant reported, dependent on age). Results showed that 13.2% of males and 18.6% of females skipped breakfast on at least one occasion, while prevalence of “regular” skipping (i.e. on both recall days) was less common (1.4% males, 3.8% females).

The adverse consequences and modifiable nature of breakfast skipping has deemed interventions to promote breakfast consumption a popular strategy to address early life inequalities across low-, middle-, and high-income countries. Despite mixed evidence regarding efficacy in achieving their intended aims [15–17], school breakfast programs, offering free or reduced cost breakfast to children and adolescents at school, have been operating in the US and the UK for decades. Historically, such initiatives have been eligibility-based but more recently have shifted toward universal models of provision to reach more students in need, representing significant financial investment. In 2019, schools provided around 2.5 billion breakfasts to children across the US, with a total investment of approximately USD 4.5billion [18]. In the UK, daily breakfast was provided to almost 300,000 children in 2018, representing a GBP 26 million investment [19].

More recently, service provision in Australia has followed suit, with not-for-profit organisations such as Foodbank, KickStart for Kids, and Red Cross delivering free breakfast in schools across the country as part of the fight against poverty and food insecurity. Considering increased investment in school breakfast programs coupled with limited evidence regarding the prevalence of breakfast skipping in Australia, it is important to understand both how many and which children and adolescents regularly skip breakfast so that interventions and policy to promote breakfast consumption can be appropriately considered.

The aim of this research was to explore the epidemiology of breakfast skipping among a contemporary, population-wide sample of children and adolescents in Australia. Specifically, we sought to identify breakfast skipping prevalence by gender, school grade, socioeconomic status, and geographical remoteness to identify among which population sub-groups breakfast skipping is most common. This research strengthens evidence regarding breakfast skipping among children and adolescents and will inform strategies for provision of school breakfast programs.

## Methods

### Data sources

This cross-sectional study used data for children and adolescents in grades 4–12 from the 2019 Wellbeing and Engagement Collection (WEC) in South Australia [20, 21]. The WEC is an annual census of student wellbeing and engagement with school, with all schools in South Australia invited to participate unlike previous Australian research limited by a lack of representative samples [7–9]. Further, information on habitual breakfast consumption, which appears to be a better predictor of later outcomes relative to reports of breakfast consumption on individual days [22], is collected from students themselves. The census is completed via an online data collection system, with time provided to students to complete the survey during school hours, usually taking between 25–45 min. The WEC measures four domains: Emotional Wellbeing, Engagement with School, Learning Readiness, and Health and Wellbeing Out of School. Each domain includes multiple constructs; some are measured using multi-item scales and others with single items [20, 21]. The current study includes data from the 2019 WEC for students from government (i.e., public) schools. The WEC was linked via a unique education identifier to South Australian Department for Education enrolment records containing information on child-level demographic characteristics. Students from non-government schools who participated in the 2019 WEC were excluded from the current study because of a lack of information on their demographic characteristics.

### Participants

Participants were grade 4–12 students (aged 8–18 years) in South Australian government schools who took part in the 2019 WEC. Overall, 88.8% of government schools participated (*n* = 453). A total of 77,322 students from these schools opted to participate, representing 67.6% of eligible students. A small number of WEC instruments (*n* = 1005, 1.3%) were invalid as students did not complete enough items. For the purpose of this analysis, students were excluded if they were over the age of 18 years (*n* = 698, 0.9%) or if they had missing data for breakfast (*n* = 4334, 5.7%) or demographic characteristic items (*n* = 597, 0.8%). Thus, the analysis sample used throughout this paper included 71,390 students who had completed data for all items in this study. The analysis sample represents 61.3% of the eligible cohort (i.e., the overall 2019 cohort of grade 4–12 students in South Australian government schools aged ≤18 years, *n* = 117,366, refer to Supplementary Table 1).

### Measures

#### Breakfast skipping

Breakfast consumption was measured within the Health and Wellbeing Out of School domain of the WEC. Students were asked “How often do you eat breakfast?” with an 8-point scale response option (Never, Once a week, 2 times a week … 6 times a week, Every day). The question is not asked with an associated timeframe and thus students respond based on their typical habits. For the purpose of this analysis, the scale was recoded into 1 = never skippers (eats breakfast every day; reference category), 2 = sometimes skippers (eats breakfast 4 to 6 days a week), 3 = often skippers (eats breakfast 1 to 3 days a week), and 4 = always skippers (never eats breakfast). Distribution of responses to the breakfast consumption item among the analysis sample, prior to categorisation for analysis, is presented in Supplementary Table 2.

#### Demographic characteristics

Demographic characteristics used in this study included gender, school grade, socioeconomic status, and geographical remoteness. Data on student gender and school grade were sourced from self-reported responses in the WEC. Gender categories included male, female or other based on students’ gender identity. Grade was categorised into the following groups: 4–5, 6–7, 8–9, and 10–12. Student residential postcode (i.e., zip code) was sourced from the Department for Education enrolment records, and based on this, community level socioeconomic status and geographical remoteness were assigned to each student. The 2016 Socio-Economic Indexes for Areas Index of Relative Socio-Economic Advantage and Disadvantage (SEIFA IRSAD) [23] provided an area level measure of socioeconomic status. Quintile 1 represents the most socioeconomically disadvantaged areas, while Quintile 5 represents the most socioeconomically advantaged areas. The 2016 Accessibility and Remoteness Index of Australia (ARIA) [24] was used as an indicator of geographical remoteness. Students were categorised to be living in Major Cities, Inner Regional, Outer Regional, Remote or Very Remote areas of South Australia. Remote and Very Remote were combined into one category due to small numbers of students in each.

### Statistical approach

WEC data and enrolment records were linked by the South Australian Department for Education. De-identified data was provided to the research team for analysis. First, demographic characteristics of the analysis sample were compared to that of the overall cohort to explore any bias in the sample used in this study. Descriptive statistics (n, %) were presented for the four breakfast skipping categories (never skippers, sometimes skippers, often skippers, and always skippers) stratified by gender, school grade, socioeconomic status and geographical remoteness. A series of multinomial logistic regression analyses were conducted to explore the relative risk ratio of sometimes, often, and always skippers compared with never skippers, according to demographic characteristics. Informed by the literature, multinomial logistic regression analyses were then conducted separately for males and females [7, 9, 10]. Analyses were conducted using Stata SE version 16 [25].

## Results

Demographic characteristics of the analysis sample were similar to that of the overall cohort, as described in Supplementary Table 1. The percentage of students living in the most socioeconomically disadvantaged areas was slightly lower in the analysis sample relative to the overall cohort (25.6% vs 28.9%), while the percentage of students living in the most socioeconomically advantaged areas was slightly higher in the analysis sample compared to the overall cohort (21.2% vs 18.6%). The analysis sample reflects trends in participation in the WEC in that a smaller proportion of students in senior grades took part in data collection [20].

Overall, 55.0% of students reported never skipping breakfast, 17.4% reported sometimes skipping, 18.0% reported often skipping, and 9.5% reported always skipping breakfast. Results in Table 1 highlight that often and always skipping breakfast were more prevalent among females than males, students in senior grades, and those living in socioeconomically disadvantaged and regional and remote areas, while differences in prevalence of sometimes skipping breakfast were less marked across all demographic variables. For example, 21.0% of females often and 10.8% always skip breakfast, compared to 15.0 and 8.1% among males, respectively, while 17.4% of both males and females reported sometimes skipping breakfast. The prevalence of always skipping breakfast among grade 10–12 students was fourfold that among grade 4–5 students (16.5% vs 4.2%), while prevalence of often skipping breakfast was more than double (26.2% vs 10.7%). Often and always skipping breakfast were most prevalent among students living in the most socioeconomically disadvantaged areas (quintile 1; 21.8 and 13.1%, respectively) compared to students living in the most socioeconomically advantaged areas (quintile 5; 13.4 and 6.1%, respectively). However, the prevalence of sometimes skipping was only about one percentage higher among students living in the most compared to the least socioeconomically disadvantaged areas (16.9% vs 18.0%, respectively). The prevalence of often and always skipping breakfast was greatest among students living in inner regional areas (19.5 and 10.6%, respectively), both of which were least prevalent among students living in major cities (17.5 and 9.2%, respectively).

**Table 1:** Prevalence of breakfast skipping (n, %) by gender, grade, socioeconomic status, and geographical remoteness among South Australian students in grades 4–12 in 2019 (*n* = 71,390)

		Never skips (*n* = 39,291, 55.0%)	Sometimes skips (*n* = 12,435, 17.4%)	Often skips (*n* = 12,858, 18.0%)	Always skips (*n* = 6806, 9.5%)
Gender	Male	21,359 (59.5)	6257 (17.4)	5385 (15.0)	2895 (8.1)
	Female	17,708 (50.7)	6082 (17.4)	7339 (21.0)	3780 (10.8)
	Other	224 (38.3)	96 (16.4)	134 (22.9)	131 (22.4)
Grade	4–5	13,952 (70.7)	2837 (14.4)	2110 (10.7)	833 (4.2)
	6–7	11,486 (60.0)	3515 (18.4)	2924 (15.3)	1235 (6.5)
	8–9	7401 (46.4)	3050 (19.1)	3487 (21.9)	2015 (12.6)
	10–12	6452 (39.0)	3033 (18.3)	4337 (26.2)	2723 (16.5)
Socioeconomic status	Quintile 1	8822 (48.3)	3087 (16.9)	3974 (21.8)	2392 (13.1)
	Quintile 2	6113 (51.9)	2088 (17.7)	2348 (19.9)	1231 (10.5)
	Quintile 3	6189 (54.7)	1976 (17.5)	2089 (18.5)	1068 (9.4)
	Quintile 4	8701 (58.5)	2554 (17.2)	2427 (16.3)	1199 (8.1)
	Quintile 5	9466 (62.6)	2730 (18.0)	2020 (13.4)	916 (6.1)
Geographical remoteness	Major Cities	27,911 (56.4)	8397 (17.0)	8645 (17.5)	4536 (9.2)
	Inner Regional	5470 (51.8)	1914 (18.1)	2056 (19.5)	1121 (10.6)
	Outer Regional	4604 (52.0)	1666 (18.8)	1680 (19.0)	910 (10.3)
	Remote/very remote	1306 (52.7)	458 (18.5)	477 (19.2)	239 (9.6)

Table 2 presents the relative risk ratio of skipping (sometimes, often, and always) compared to never skipping breakfast, according to demographic characteristics. The relative risk ratio of sometimes skipping compared to never skipping breakfast was 1.17 (95% CI 1.13–1.22) times higher for females compared to males, while the relative risk ratio of often skipping was 1.64 (95% CI 1.58–1.71) times higher, and always skipping was 1.57 (95% CI 1.49–1.66) times higher, compared to never skipping breakfast. Students in grade 10–12 had a 2.31 (95% CI 2.18–2.45) times greater risk of sometimes skipping, a 4.44 (95% CI 4.19–4.72) times greater risk of often skipping, and a 7.07 (95% CI 6.51–7.68) times greater risk of always skipping breakfast, compared to never skipping, relative to students in grade 4–5. Students living in the most socioeconomically disadvantaged areas had 1.21 (95% CI 1.14–1.29) times the risk of sometimes skipping, 2.11 (95% CI 1.99–2.24) times the risk of often skipping, and 2.80 (95% CI 2.58–3.04) times the risk of always skipping breakfast, compared to never skipping, relative to those living in the most socioeconomically advantaged areas. Students living in inner regional areas had 1.16 (95% CI 1.10–1.23) times the risk of sometimes skipping, 1.21 (95% CI 1.15–1.28) times the risk of often skipping, and 1.26 (95% CI 1.17–1.35) times the risk of always skipping, compared to never skipping, relative to students living in major cities.

**Table 2:** Multinomial logistic regression results: Prevalence of breakfast skipping (relative risk ratio) by gender, grade, socioeconomic status, and geographical remoteness among South Australian students in grades 4–12 in 2019 (*n* = 71,390)

		Sometimes skips		Often skips		Always skips	
		RRR (95% CI)	*P*	RRR (95% CI)	*P*	RRR (95% CI)	*P*
Gender	Male	ref	–	ref	–	ref	–
	Female	1.17 (1.13–1.22)	<0.001	1.64 (1.58–1.71)	<0.001	1.57 (1.49–1.66)	<0.001
	Other	1.46 (1.15–1.86)	0.002	2.37 (1.91–2.95)	<0.001	4.31 (3.47–5.37)	<0.001
Grade	4–5	ref	–	ref	–	ref	–
	6–7	1.50 (1.42–1.59)	<0.001	1.68 (1.58–1.79)	<0.001	1.80 (1.64–1.97)	<0.001
	8–9	2.03 (1.91–2.15)	<0.001	3.12 (2.93–3.31)	<0.001	4.56 (4.19–4.97)	<0.001
	10–12	2.31 (2.18–2.45)	<0.001	4.44 (4.19–4.72)	<0.001	7.07 (6.51–7.68)	<0.001
Socioeconomic status	Quintile 1	1.21 (1.14–1.29)	<0.001	2.11 (1.99–2.24)	<0.001	2.80 (2.58–3.04)	<0.001
	Quintile 2	1.18 (1.11–1.26)	<0.001	1.80 (1.68–1.93)	<0.001	2.08 (1.90–2.28)	<0.001
	Quintile 3	1.11 (1.04–1.18)	0.003	1.58 (1.48–1.69)	<0.001	1.78 (1.62–1.96)	<0.001
	Quintile 4	1.02 (0.96–1.08)	0.573	1.31 (1.22–1.40)	<0.001	1.42 (1.30–1.56)	<0.001
	Quintile 5	ref	–	ref	–	ref	–
Geographical remoteness	Major Cities	ref	–	ref	–	ref	–
	Inner Regional	1.16 (1.10–1.23)	<0.001	1.21 (1.15–1.28)	<0.001	1.26 (1.17–1.35)	<0.001
	Outer Regional	1.20 (1.13–1.28)	<0.001	1.18 (1.11–1.25)	<0.001	1.22 (1.13–1.31)	<0.001
	Remote/Very Remote	1.17 (1.05–1.30)	0.006	1.18 (1.06–1.31)	0.003	1.13 (0.98–1.30)	0.100

*RRR* Relative risk ratio, *CI* Confidence interval

When exploring the likelihood of skipping versus never skipping breakfast among males and females separately (see Tables 3 and 4), results show a steeper grade level gradient among females compared to males. Females in grade 10–12 had 2.65 (95% CI 2.43–2.89) times the risk of sometimes skipping, 5.79 (95% CI 5.32–6.30) times the risk of often skipping, and 10.03 (95% CI 8.89–11.32) times the risk of always skipping, compared to never skipping, than females in grade 4–5. The relative risk ratio of sometimes (2.08, 95% CI 1.92–2.26), often (3.43, 95% CI 3.15–3.74) and always (5.02, 95% CI 4.47–5.64) skipping breakfast, compared to never skipping, among males in grade 10–12 were not as high, relative to those in grade 4–5. There were no clear differences in the likelihood of skipping breakfast by socioeconomic disadvantage or geographical remoteness between females and males.

**Table 3:** Multinomial logistic regression results: Prevalence of breakfast skipping (relative risk ratio) by grade, socioeconomic status, and geographical remoteness among male South Australian students in grades 4–12 in 2019 (*n* = 35,896)

		Sometimes skips			Often skips			Always skips		
		n (%)	RRR (95% CI)	*P*	n (%)	RRR (95% CI)	*P*	n (%)	RRR (95% CI)	*P*
Grade	4–5	1448 (14.4)	ref	–	1027 (10.2)	ref	–	444 (4.4)	ref	–
	6–7	1722 (18.0)	1.39 (1.28–1.50)	<0.001	1181 (12.3)	1.34 (1.22–1.47)	<0.001	526 (5.5)	1.38 (1.21–1.57)	<0.001
	8–9	1524 (19.1)	1.73 (1.60–1.88)	<0.001	1349 (16.9)	2.16 (1.98–2.37)	<0.001	770 (9.6)	2.86 (2.53–3.23)	<0.001
	10–12	1563 (18.9)	2.08 (1.92–2.26)	<0.001	1828 (22.1)	3.43 (3.15–3.74)	<0.001	1155 (14.0)	5.02 (4.47–5.64)	<0.001
Socio-economic status	Quintile 1	1586 (17.3)	1.25 (1.15–1.35)	<0.001	1715 (18.7)	2.15 (1.97–2.36)	<0.001	1033 (11.3)	2.90 (2.56–3.29)	<0.001
	Quintile 2	1107 (18.7)	1.29 (1.17–1.41)	<0.001	993 (16.8)	1.84 (1.67–2.04)	<0.001	539 (9.1)	2.24 (1.95–2.57)	<0.001
	Quintile 3	952 (17.0)	1.07 (0.97–1.17)	0.160	839 (14.9)	1.51 (1.36–1.67)	<0.001	442 (7.9)	1.78 (1.54–2.05)	<0.001
	Quintile 4	1275 (16.8)	1.01 (0.93–1.10)	0.794	1002 (13.2)	1.27 (1.15–1.41)	<0.001	507 (6.7)	1.44 (1.25–1.65)	<0.001
	Quintile 5	1337 (17.5)	ref	–	836 (11.0)	ref	–	374 (4.9)	ref	–
Geographical remoteness	Major Cities	4192 (16.8)	ref	–	3628 (14.5)	ref	–	1942 (7.8)	ref	–
	Inner Regional	968 (18.5)	1.19 (1.10–1.29)	<0.001	835 (16.0)	1.18 (1.09–1.29)	<0.001	465 (8.9)	1.23 (1.10–1.37)	<0.001
	Outer Regional	871 (19.7)	1.30 (1.19–1.41)	<0.001	723 (16.4)	1.24 (1.14–1.36)	<0.001	381 (8.6)	1.22 (1.09–1.38)	0.001
	Remote/Very Remote	226 (18.5)	1.20 (1.03–1.40)	0.022	199 (16.3)	1.22 (1.04–1.43)	0.017	107 (8.8)	1.22 (0.99–1.51)	0.058

*RRR* Relative risk ratio, *CI* Confidence interval

**Table 4:** Multinomial logistic regression results: Prevalence of breakfast skipping (relative risk ratio) by grade, socioeconomic status, and geographical remoteness among female South Australian students in grades 4–12 in 2019 (*n* = 34,909)

		Sometimes skips			Often skips			Always skips		
		n (%)	RRR (95% CI)	*P*	n (%)	RRR (95% CI)	*P*	n (%)	RRR (95% CI)	*P*
Grade	4–5	1374 (14.3)	ref	–	1072 (11.2)	ref	–	381 (4.0)	ref	–
	6–7	1770 (18.7)	1.65 (1.52–1.78)	<0.001	1717 (18.2)	2.05 (1.88–2.23)	<0.001	687 (7.3)	2.31 (2.03–2.63)	<0.001
	8–9	1494 (19.2)	2.46 (2.26–2.67)	<0.001	2089 (26.9)	4.40 (4.04–4.79)	<0.001	1196 (15.4)	7.09 (6.27–8.02)	<0.001
	10–12	1444 (17.8)	2.65 (2.43–2.89)	<0.001	2461 (30.4)	5.79 (5.32–6.30)	<0.001	1516 (18.7)	10.03 (8.89–11.32)	<0.001
Socio-economic status	Quintile 1	1479 (16.6)	1.20 (1.10–1.31)	<0.001	2214 (24.8)	2.10 (1.94–2.29)	<0.001	1316 (14.7)	2.81 (2.52–3.14)	<0.001
	Quintile 2	966 (16.7)	1.10 (1.00–1.21)	0.057	1336 (23.1)	1.78 (1.62–1.95)	<0.001	676 (11.7)	2.03 (1.79–2.29)	<0.001
	Quintile 3	1013 (18.0)	1.16 (1.06–1.27)	0.002	1229 (21.9)	1.65 (1.50–1.81)	<0.001	600 (10.7)	1.81 (1.60–2.06)	<0.001
	Quintile 4	1257 (17.5)	1.03 (0.95–1.13)	0.474	1395 (19.4)	1.34 (1.23–1.47)	<0.001	670 (9.3)	1.45 (1.29–1.64)	<0.001
	Quintile 5	1367 (18.5)	ref	–	1165 (15.8)	ref	–	518 (7.0)	ref	–
Geographical remoteness	Major Cities	4134 (17.2)	ref	–	4925 (20.5)	ref	–	2507 (10.4)	ref	–
	Inner Regional	933 (17.8)	1.14 (1.05–1.24)	0.002	1199 (22.9)	1.23 (1.14–1.33)	<0.001	629 (12.0)	1.27 (1.15–1.40)	<0.001
	Outer Regional	785 (17.9)	1.11 (1.02–1.21)	0.019	942 (21.5)	1.12 (1.03–1.21)	0.007	517 (11.8)	1.21 (1.09–1.34)	<0.001
	Remote/Very Remote	230 (18.5)	1.14 (0.97–1.33)	0.108	273 (22.0)	1.13 (0.98–1.31)	0.097	127 (10.2)	1.03 (0.85–1.26)	0.735

*RRR* Relative risk ratio, *CI* Confidence interval

## Discussion

This study explored the prevalence of breakfast skipping among a contemporary, population-wide sample of grade 4–12 students (aged 8–18 years) in Australia. Encouragingly, the majority of students reported never skipping breakfast. However, about 1 in 3 students reported skipping breakfast sometimes or often, and 1 in 10 reported skipping breakfast every day. Skipping breakfast was more prevalent among females, students in senior grades, and those living in socioeconomically disadvantaged and regional and remote areas. Overall, results indicate that breakfast skipping among children and adolescents is considerably more prevalent than previous research (focused on ages 2–17 years) in Australia suggests [12–14].

An increase in breakfast skipping over the past decade may reflect shifts in family structures and/or routines as well as an increased focus on diet culture among young people. Although it is possible that our results, in part, reflect an increase in the prevalence of breakfast skipping over time, it is important to consider differences in how breakfast consumption in previous studies was measured, how breakfast skipping was defined, as well as sample differences. For instance, the 2011–12 National Nutrition and Physical Activity Survey measured breakfast consumption on two days (recalled by caregivers or participants, dependent on age), with breakfast defined as an eating occasion of 210 kJ or more called ‘breakfast’ by participants. The study was considered nationally representative with a total sample of 1592 participants aged 2–17 years with an overall participation rate of 77% completing nutrition diaries. Although population weighted, as common with such survey methodology, the sample was biased towards the higher socioeconomic [26]. The current study measured habitual breakfast consumption (i.e., in a typical week) reported by children and adolescents themselves, though the WEC did not provide respondents with a definition of breakfast. The WEC employed a census approach, aiming to collect data from all children and adolescents aged 8 to 18 years with a total sample of 71,390 from government schools in South Australia. On average, South Australia is more socioeconomically disadvantaged than Australia overall, with 26% of the population living in the poorest areas (i.e. SEIFA quintile 1, vs 20% in Australia) [27].

Prevalence of breakfast skipping reported in the current study exceeds that of international evidence also. A recent systematic review (n = 285,626, aged 2–18 years) including 39 studies across 33 countries concluded that the majority of research reported between 10–30% of children and adolescents skipped breakfast [7]. Authors highlighted great variability in breakfast skipping prevalence across studies however, ranging from 0.7 to 74.7%, which was dependent on the various measures and definitions of breakfast skipping used.

Aligned with previous research [2, 7, 9–11], results from the current study indicate breakfast skipping was more prevalent among females, students in senior grades (i.e., adolescents), and those living in socioeconomically disadvantaged and regional and remote areas. A systematic review exploring family/household factors associated with breakfast skipping among 6–18-year-olds highlighted that living in a single parent/caregiver home was among the factors with strongest evidence of an association with children and young people skipping breakfast [28]. This supports the notion that family structures and/or routines influence breakfast consumption, and that changes in family structures and/or routines over time may be contributing to a higher prevalence of breakfast skipping among children and adolescents over time.

Analyses disaggregated by gender add to existing evidence by highlighting that grade level gradients in breakfast skipping were more marked among females than males. Indeed, breakfast skipping was most prevalent among females in grade 10–12, with almost 1 in 5 skipping breakfast every day. Previous researchers have theorised that gender differences in breakfast consumption may reflect greater body image and/or dieting concerns among adolescent females, relative to males. However, research exploring the predictors of breakfast consumption among Australian adolescents (*n* = 481; aged 11–18 years) found breakfast skipping due to weight control reasons relatively uncommon (4%), though reasons for skipping breakfast were not reported separately for males and females [8]. Conversely, there were no clear differences in the likelihood of skipping breakfast by socioeconomic disadvantage or geographical remoteness between females and males.

### Implications

Findings highlight several key considerations for policy and practice that seeks to promote breakfast consumption. Provision of breakfast to students in education settings, indeed school feeding programs more broadly, are implemented for a variety of purposes. In low- and middle-income countries, education is typically non-mandatory and thus school feeding seeks to promote school attendance. In high-income countries including Australia, school breakfast programs aim to ensure all students have access to a nutritious breakfast with the goal of promoting engagement with learning and ultimately improving academic outcomes.

Importantly, findings provide insight into the magnitude of the problem. That is, the number and proportion of school-aged children and adolescents who do not regularly eat breakfast every day and may benefit from some form of support or intervention to promote breakfast consumption. Despite considerable investment in school breakfast provision across the country (see for example [29, 30]), results indicate a substantial proportion of children and adolescents regularly skip breakfast, suggesting alternative supports are required.

Although the influence of socioeconomic disadvantage is clear, evidently it is not the sole driver of breakfast skipping. Rather, a variety of factors are at play as it is not only students living in the poorest areas who skip breakfast. School breakfast programs are generally targeted at socioeconomically disadvantaged communities. Findings from international research on the impacts of school breakfast programs on school attendance, student wellbeing, academic performance, nutritional intake, and physical health, are mixed [15–17]. What is clear is that promoting attendance at school breakfast programs, particularly attendance among the students who might need it most (i.e. students who skip breakfast), is a challenge [31]. Together, evidence suggests the need to explore drivers of breakfast skipping, which are likely to differ across population sub-groups, to improve strategies to promote breakfast consumption. Schools and education systems more broadly have the potential to provide children and young people with environments that nurture healthy habits. When it comes to nutrition including breakfast consumption, this could extend beyond meal provision to education or health promotion efforts focused on shifting attitudes toward breakfast consumption.

### Limitations

A key strength of this study lies in that it is, to our knowledge, the first to explore breakfast skipping prevalence among a large, population-wide sample of students in Australia. While the analysis sample is large, the results reported are limited to children and adolescents attending government (public) schools. In South Australia, about two thirds of children and adolescents attend government schools and these students are generally more socioeconomically disadvantaged than those attending non-government schools [32, 33]. As such, our results are likely to over-represent the more disadvantaged children and adolescents in South Australia. Similarly, when generalising these results to Australia overall, the prevalence data reported in this study are likely to over-represent the more disadvantaged students.

The study lacked information regarding breakfast content and reasons behind breakfast skipping that are important when considering implications for policy and practice. For instance, some students who reported eating breakfast may consider a beverage or a sugar-filled snack to be their breakfast. Although the objective of this study was to explore prevalence of breakfast consumption vs non-consumption, it is important to note that the percentage of children and adolescents who consumed a healthy, nutritious breakfast cannot be deduced from results. As such, when considering the prevalence figures reported here they are likely to over-represent students consuming a breakfast that is healthy and nutritious. Further, as mentioned, the reasons behind why students skipped breakfast may vary from lack of time in the morning, to food insecurity, to challenges surrounding body image. Strategies to promote breakfast consumption in light of each of these drivers would likely differ. Therefore, the “why” is key in shaping supports to reduce the prevalence of breakfast skipping. Additional information of this nature would allow investigation beyond breakfast skipping prevalence alone to explore how many and which students are not consuming a healthy breakfast, and why, both of which are important questions for future research.

## Conclusions

The prevalence of breakfast skipping among children and adolescents appears considerably more prevalent than previous studies suggest. Building on existing research that has been limited by small, non-representative samples, findings from the current study suggest alternative supports are required to ensure all children and adolescents regularly eat breakfast. To be effective, strategies to promote breakfast consumption need to be informed by an understanding of the drivers of breakfast skipping across population sub-groups.

## Supplementary Information

**Additional file 1.**

**Additional file 2.**

## Data Availability

Approval by an accredited research ethics committee is required to access the WEC data for research purposes. Access to data used in this study can be requested from the data custodian, the South Australian Department for Education (DfE) website (www.education.sa.gov.au) or via email (education.researchunit@sa.gov.au).

## Abbreviations

ARIA: Accessibility and Remoteness Index of Australia
CI: Confidence interval
DfE: Department for Education
RRR: Relative risk ratio
SEIFA IRSAD: Socio-Economic Indexes for Areas - Index of Relative Socio-Economic Advantage and Disadvantage
WEC: Wellbeing and Engagement Collection
