# Supplementary material for: Prevalence of breakfast skipping among children and adolescents: a cross-sectional population level study
Source: BMC Pediatr. 2022 Apr 23;22:220. doi: 10.1186/s12887-022-03284-4 (PMC9034546; doi:10.1186/s12887-022-03284-4)
Supplement: Supplementary file 1 — Additional file 1. [file 12887_2022_3284_MOESM1_ESM.docx]

Supplementary Table 1. Demographic characteristics (n, %) of the analysis sample and the overall cohort

|  | | Analysis sample  (n = 71,390) | Overall cohort  (n = 117,366) |
| --- | --- | --- | --- |
| Gender | Male | 35,896 (50.3) | 60,268 (51.4) |
|  | Female | 34,909 (48.9) | 56,465 (48.1) |
|  | Other | 585 (0.8) | 633 (0.5) |
| Grade | 4-5 | 19,732 (27.6) | 28,976 (24.7) |
|  | 6-7 | 19,160 (26.8) | 27,287 (23.3) |
|  | 8-9 | 15,953 (22.4) | 24,450 (20.8) |
|  | 10-12 | 16,545 (23.2) | 36,653 (31.2) |
| Socioeconomic status | Quintile 1 | 18,275 (25.6) | 34,005 (29.0) |
|  | Quintile 2 | 11,780 (16.5) | 19,357 (16.5) |
|  | Quintile 3 | 11,322 (15.9) | 18,511 (15.8) |
|  | Quintile 4 | 14,881 (20.8) | 22,568 (19.2) |
|  | Quintile 5 | 15,132 (21.2) | 21,932 (18.7) |
|  | Missing | 0 | 993 (0.9) |
| Geographical remoteness | Major Cities | 49,489 (69.3) | 80,101 (68.3) |
|  | Inner Regional | 10,561 (14.8) | 17,068 (14.5) |
|  | Outer Regional | 8,860 (12.4) | 14,782 (12.6) |
|  | Remote/Very Remote | 2,480 (3.5) | 4,439 (3.8) |
|  | Missing | 0 | 976 (0.8) |
