# Supplementary material for: Prevalence of breakfast skipping among children and adolescents: a cross-sectional population level study
Source: BMC Pediatr. 2022 Apr 23;22:220. doi: 10.1186/s12887-022-03284-4 (PMC9034546; doi:10.1186/s12887-022-03284-4)
Supplement: Supplementary file 2 — Additional file 2. [file 12887_2022_3284_MOESM2_ESM.docx]

Supplementary Table 2. Distribution (n, %) of responses to breakfast consumption item among analysis sample (n = 71,390)

|  | How often do you eat breakfast? | |
| --- | --- | --- |
|  | n | % |
| Never | 6,806 | 9.5 |
| Once a week | 4,761 | 6.7 |
| Twice a week | 4,118 | 5.8 |
| 3 times a week | 3,979 | 5.6 |
| 4 times a week | 3,386 | 4.7 |
| 5 times a week | 4,287 | 6.0 |
| 6 times a week | 4,762 | 6.7 |
| Everyday | 39,291 | 55.0 |
